# The Sas3p and Gcn5p histone acetyltransferases are recruited to similar genes

**DOI:** 10.1186/gb-2007-8-6-r119

**Published:** 2007-06-20

**Authors:** Lorena E Rosaleny, Ana B Ruiz-García, José García-Martínez, José E Pérez-Ortín, Vicente Tordera

**Affiliations:** 1 Departament de Bioquímica i Biologia Molecular, Universitat de València, València. Spain; 2 Laboratori de Chips de DNA del Servei Central de Suport a la Investigació Experimental, Universitat de València, València

## Abstract

**Background:**

Specific histone modifications can perform several cellular functions, for example, as signals to recruit trans-acting factors and as modulators of chromatin structure. Acetylation of Lys14 of histone H3 is the main target of many histone acetyltransferases *in vitro *and may play a central role in the stability of the nucleosome. This study is focused on the genome-wide binding of *Saccharomyces cerevisiae *histone acetyltransferases that are specific for Lys14 of histone H3.

**Results:**

We have used a variation of the genome-wide location analysis method, based on a macroarray platform, to identify binding sites of yeast histone acetyltransferase catalytic subunits and to correlate their positions with acetylation of Lys14 of histone H3. Our results revealed that the histone acetyltransferases Sas3p and Gcn5p are recruited to a pool of intensely transcribed genes and that there is considerable overlap between the two cohorts of Sas3p and Gcn5p bound gene pools. We also demonstrate a positive correlation between binding sites of both proteins and the acetylation state of Lys14 of histone H3. Finally, a positive correlation between the decrease of H3 Lys14 acetylation in a *GCN5 *deleted strain and the Gcn5p genome occupancy is shown.

**Conclusion:**

Our data support a model in which both Gcn5p and Sas3p act as general activators of an overlapping pool of intensely transcribed genes. Since both proteins preferentially acetylate Lys14 of histone H3, our data support the hypothesis that acetylation of this specific residue facilitates the action of the transcriptional apparatus.

## Abstract 2

A macroarray platform was used to identify binding sites of yeast histone acetyltransferase catalytic subunits and to correlate their positions with acetylation of lysine 14 of histone H3, revealing that Sas3p and Gcn5p are recruited to similar sets of intensely transcribed genes.

## Background

Most of the protein mass in eukaryotic chromatin is composed of histones, which are crucial for the organization of the highly conserved nucleosomal structure. Histones are subjected to a wide variety of post-translational modifications, most of them reversible [1,2], that can influence chromatin functions by different mechanisms. This epigenetic information, composed of histone modifications, has been called the histone code [3-6] and can be considered as specific binding surfaces for the recruitment of activators or repressors of biological processes. While the term code has raised some controversy, there is growing evidence suggesting that histone modifications act as recognition signals for proteins that regulate gene expression and other chromatin functions [7]. The existence of the histone code does not exclude the hypothesis that several histone modifications may also directly affect the nucleosomal structure or the folding properties of the chromatin, facilitating the action of the protein complexes that regulate expression, repair, recombination and other essential functions of the eukaryotic genome.

Several types of enzymatic activities, generically referred to as chromatin regulators [8], can carry out the modifications of chromatin structure. The best-studied group is that of the histone acetyltransferases (HATs). These enzymes acetylate specific lysine residues, most of which are localized in the amino-terminal tail of the histones, and hence are responsible for many of the signals of the histone code. Nine different proteins that exhibit HAT activity *in vitro *have been described to date in *Saccharomyces cerevisiae*: Gcn5p, Hat1p, Elp3p, Hpa2p, Esa1p, Sas2p, Sas3p, Nut1p and Taf1p. Some of the properties of this set of enzymes are consistent with the histone code hypothesis. For instance, they are able to selectively acetylate particular histone residues. Moreover, most of them have physical and functional connections to transcriptional regulation and act as complexes composed of several subunits, which can modulate their activities. Nevertheless, there are also some features of these enzymes that are hard to reconcile with their proposed role in a combinatorial signaling system, which involves four different histones, each one with several lysine targets. Thus, with the data available to date, if we omit Hat1p [9] and Esa1p [10,11], which selectively modify histone H4, the remainder of the HATs have a marked preference, at least *in vitro*, for histone H3, and particularly Lys14 (H3K14). Hence, there seems to be something special about this position because most HATs have potential H3K14 acetylation (H3K14ac) activity, exclusively or accompanied by other specificities. More specifically, it has been reported that Gcn5p [12-14], Sas3p [15], Hpa2p [16], Elp3p [17] and Taf1p [18] preferentially acetylate H3K14 *in vitro*. Nut1p also preferentially acetylates H3, but its specificity is unknown. Finally, the SAS complex, which is composed of the Sas2p acetylase bound to the Sas4 and Sas5 proteins, acetylates free H3 at Lys14 [19], although its specificity for nucleosome-assembled histones seems to be limited to H4 [20].

Despite the important role that acetylation of H3K14 could play, the HATs responsible for this modification *in vivo *are not well known. There are some studies where the acetylation state of bulk H3 obtained from different HAT mutant strains has been analyzed. However, most of these studies used an antibody specific for histone H3 acetylated at both K9 and K14 and, hence, it is not possible to determine if a lower signal in histone H3 isolated from the mutant strains is the consequence of a decrease in the acetylation state of H3K9, H3K14 or both. For instance, the acetylation analysis of a *gcn5*Δ strain did not definitively show which position was affected [15,21,22]. For *sas3*Δ [15,21], *elp3*Δ [21,22] or *hpa2*Δ [21] strains no differences were observed when comparing the relative amounts of H3K14ac in the mutant and the wild-type strain. When site-specific antibodies were used in a *gcn5*Δ strain the results showed that Gcn5p likely acetylates all sites in H3 with the sole exception of H3K14 [23]. These authors commented on the striking contradiction between this finding and the very strong preference of Gcn5p for acetylating H3K14 *in vitro*. On the other hand, using chromatin immunoprecipitation of histones and DNA-microarray analysis (ChIP-chip), a positive association between Gcn5p occupancy and transcriptional activity [8,24] and between Gcn5p occupancy and acetylation of H3K14 has been found [24], suggesting an active role for this HAT in the *in vivo *acetylation of H3K14 and transcriptional activation. Moreover, it was reported that acetylation of H3K14 peaks at the start sites of active genes and drops substantially across the open reading frames (ORFs) on a genome-wide level [24]. Similar results were obtained using a high-resolution tiling microarray with single-nucleosome resolution, covering 0.5 megabases of yeast genomic sequence [25]. These observations suggest that this H3 modification may directly facilitate the action of the transcriptional apparatus, at least at the initiation step.

In this study, we have investigated the genome-wide binding of known *Saccharomyces cerevisiae *HATs that have shown specificity for H3K14. With this aim, we have developed a ChIP-chip method based on the use of macroarrays. Using this technique, we have shown that Sas3p, like Gcn5p, is generally recruited to a similar pool of actively transcribed protein-coding genes. We also have shown that Gcn5p and Sas3p are involved in the acetylation state of H3K14 *in vivo*. Additionally, we found a relationship between Sas2p and double-strand break repair and ubiquitin-specific protease activity.

## Results and discussion

### Testing the protocol

Since the development of ChIP-chip technology, this method has been extensively used to analyze the histone modification patterns of chromatin and the binding sites of histone-modifying enzymes in yeast. These studies employ microarrays to hybridize the PCR-amplified, immunoprecipitated DNA. We have developed a method of ChIP-chip (see Materials and methods) based on the utilization of membrane-based DNA macroarrays. Macroarrays, while less widespread than microarrays, are also used by many laboratories and offer an interesting alternative. Macroarrays can be hybridized many times, up to ten times, without a measurable reduction in their performance [26], thus greatly reducing the cost of the experiments. The nature of DNA present in chips utilized in chromatin modification studies is variable. Most of the procedures employ microarrays containing ORFs, intergenic regions (IGRs), or both, from the yeast genome. There are also DNA microarrays that use oligonucleotide probes, which cover most of the yeast genome [24], and high-density oligonucleotide microarrays with approximately 20 base-pair (bp) resolution, which cover a small percentage of the yeast genome [25].

**Figure 1:**
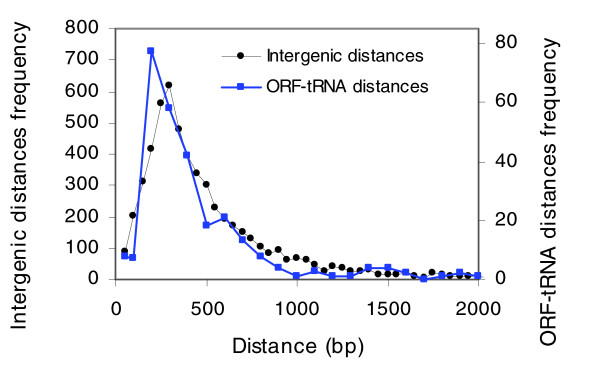
Distribution of intergenic (coding or non-coding genes) and ORF-tRNA distances. The frequency of every intergenic distance in *S. cerevisisae *is plotted [31]. Coordinates of all tRNA genes were collected and distances from adjacent genes were calculated. The frequency of each distance is plotted.

The macroarrays used in this study are made with ORF DNA, 93% of which are complete ORFs [26]. ORF macroarrays could be compatible with analyzing genome-wide protein binding if the DNA samples obtained from the immunoprecipitation are long enough to overlap ORF probes [27]. To determine the probability of detecting protein binding at different distances from an ORF, we determined the genome-wide location of Rpc160, a subunit of the RNA polymerase III from *S. cerevisiae*. There were several reasons for this selection. First, class III genes in yeast are well known and they have been studied by three independent ChIP-chip studies using different RNA polymerase III subunits [28-30]. RNA polymerase III transcribes a large set of genes encoding untranslated RNAs. Genes transcribed by this polymerase included 275 tRNA genes and six other small RNAs (for simplicity they were not analyzed in this study). We considered 275 to be a statistically relevant number of cases and, in any case, much higher than would be feasible by conventional ChIP experiments. Second, tRNA promoters are intragenic and the average gene size is only 80 bp. These facts allow for high resolution estimates of the range of distances of the identified RNA polymerase III interactions. Finally, we performed an *in silico *analysis of the positioning of the 275 tRNA genes and found that they are randomly distributed compared to distances between ORFs [31] in the yeast genome (Figure 1). Both distributions have a common trend and it can be assumed, therefore, that the detection of RNA polymerase III binding to tRNAs may mimic the detection of the binding of any protein to DNA.

Among the 275 tRNA genes, we focused on the 179 tRNA genes that are at less than 400 bp from an ORF because IGRs in yeast showed a distribution with a maximum at lengths from 150 to 800 bp (Figure 1). Therefore, the middle points of the majority of IGRs are at a distance of less than 400 bp from an ORF. Moreover, it has been described that binding sites for transcriptional regulators in yeast are not uniformly distributed over the promoter regions, but present a sharp peak around 180 bp upstream of the transcription start site [32]. Analysis of the macroarrays showed ORFs that are significantly enriched in the immunoprecipitated samples. The measurement of the significance level for each ORF using a z-test for independent data (see Materials and methods) identified 112 ORFs, 89 of which correspond to tRNA-detecting ORFs. In all cases we assigned the most proximal tRNA to each ORF. As expected, the percentage of tRNAs detected increases when the distance between the tRNA and the ORF decreases (Table 1). Complete data sets are available in Additional data file 1. With the method described here, we detected 100% of the tRNAs overlapping ORFs and 86% of the tRNAs at less than 100 bp (IGR of 200 bp) from an ORF. The resolution of our ChIP-chip method has been established experimentally by determining the distance range and the percentage of success of macroarray probes in identifying an immunoprecipitated DNA sequence in yeast. It seems likely that this resolution can also be applicable to other ChIP-chip experiments.

**Table 1:** Percentage of tRNAs detected over a range of distances from an adjacent ORF

Distance tRNA-ORF	Total no. of tRNAs	Detected tRNAs	Percentage of detected tRNAs	Accumulated percentage of detected tRNAs
Overlapping	8	8	100	-
1-100 bp	7	6	86	86
101-200 bp	72	43	60	64
201-300 bp	53	17	32	50
301-400 bp	39	9	23	44

### Sas3p and Gcn5p are recruited to a similar set of active genes

**Figure 2:**
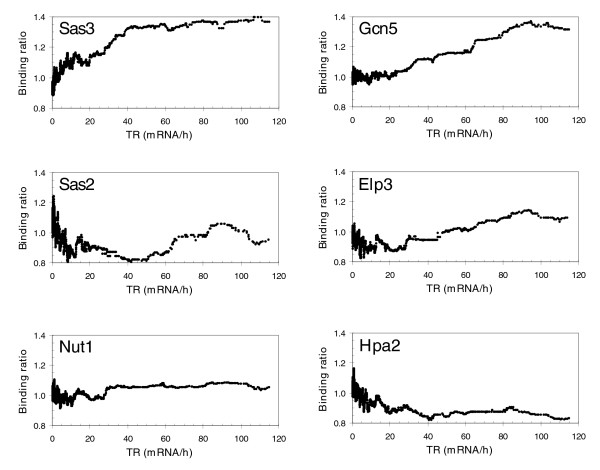
Correlations between several histone acetyltransferase occupancies and transcription rates. The occupancy was obtained by calculating the moving median of the binding ratio over a sliding window of 100 genes across all genes ordered by transcription rate. The binding ratio was calculated as the ratio of chromatin-immunoprecipitated signals between the epitope-tagged strain and the untagged parent, using the same antibodies. Transcription rate data [36] were also transformed as a moving median of values for each gene over a sliding window of 100 genes across all of them.

We analyzed the genome-wide location of all HATs previously described as histone H3 acetyltransferases *in vitro *or *in vivo*: Gcn5p, Elp3p, Hpa2p, Sas3p, Sas2p and Nut1p. Taf1p was first described as a free histone H3 HAT through *in vitro *HAT assays. However, it has recently been reported that yeast Taf1p is not a major physiological HAT of bulk H3 histones [21] and, hence, it was not analyzed in this study. Of the HATs analyzed, only Elp3p [33] and Gcn5p [8,24] were previously studied on a genome-wide scale. In the first case, Elp3p was not detected under any conditions tested and further analysis showed that this protein is predominantly located in the cytoplasm [33]. In contrast, genome-wide location analysis of Gcn5p revealed that occupancy of protein-coding genes by this protein correlates with transcription rates [8,24]. The correlation between binding trend and transcription rates has been extensively used by other groups [34,35]. Consistent with this criterion, we investigated the correlation between HAT occupancy and transcription rates. Our results indicate that genome-wide occupancy of protein-coding genes by both Sas3p and Gcn5p positively correlates with transcription rates (Figure 2; Additional data files 2-7). Nevertheless, the other four HATs analyzed did not show this sort of correlation. The results obtained for Gcn5p not only confirm previously reported data using DNA microarrays [8], but also validate our method to investigate genome-wide location of chromatin regulators that normally bind promoters. However, the most important finding of these experiments is that Sas3p showed a binding pattern similar to that of Gcn5p. These results provide the first evidence showing that Sas3p is recruited to intensely transcribed genes, similar to Gcn5p. It is important to indicate that the positive correlation between transcription rate and occupancy for Sas3p and Gcn5p was obtained not only when we used transcription rate data normally employed by other authors [36], but also when we used the more precise transcription rate data obtained from genomic run-on experiments [37] (results not shown).

**Figure 3:**
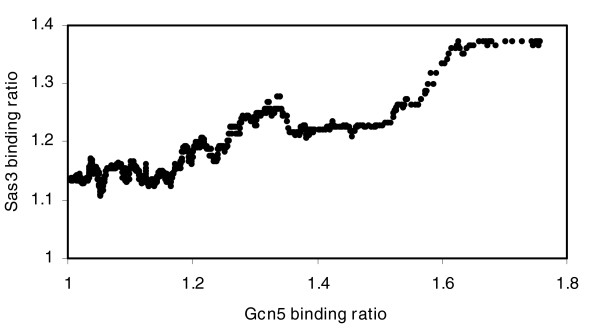
Correlation between Sas3p and Gcn5p histone acetyltransferase occupancies. The occupancy of both HATs was calculated as in Figure 2. Only ORFs that showed binding ratios higher than one were plotted. Sas3p occupancies were plotted as a function of Gcn5p occupancies and a correlation coefficient (r) of 0.91 was obtained.

When we analyzed genes bound by Sas3p and Gcn5p, we used the same criteria as those previously described by other authors for chromatin regulators [8]. The Gene Ontology (GO) categories of genes bound by Gcn5p and Sas3p with a binding ratio ≥1.5 showed, as expected, categories of highly transcribed genes (Table 2). More interestingly, the GO categories obtained for both proteins are practically identical; moreover, these remain the same in the 47 genes bound by both Gcn5p and Sas3p at binding ratios ≥1.5 (Table 2). We analyzed the number of common genes bound by the two proteins at different binding ratios compared to that expected at random. The results show that the higher the threshold of the binding ratio, the higher the coincidence between the two activator HATs, reaching up to a ten-fold increase relative to the overlap expected at random (*P *value 3.8 × 10^-13^) for a binding ratio ≥1.8 (results not shown). Moreover, when we compared the Sas3p and Gcn5p occupancies related to specific ORFs, a positive correlation was observed (Figure 3), indicating that both HATs are recruited to similar active genes. This finding is important because Sas3p, the catalytic subunit of NuA3 HAT complex [38], has been implicated in transcriptional silencing [39] and our results provide the first evidence that Sas3p could act as a transcriptional activator, like Gcn5p. In agreement with the hypothesis, it was reported that among the possible double disruptions involving *HPA2*, *SAS2*, *SAS3 *or *GCN5*, only the combination of *sas3*Δ*gcn5*Δ was synthetically lethal, due to the loss of acetyltransferase activity [15]. This result suggests that Gcn5p and Sas3p might have overlapping roles in histone acetylation that are essential for cell cycle progression or other important cellular functions [15]. Thus, in the absence of *GCN5*, Sas3p is essential for histone acetylation. Our data support a model in which both Gcn5 and Sas3 proteins act as general activators of a largely overlapping pool of intensely transcribed genes.

**Table 2:** Genes bound by Gcn5p and Sas3p

GO attribute	No. of genes in category	No. of genes bound by Gcn5p (*P *value)	No. of genes bound by Sas3p (*P *value)	No. of genes bound by both (*P *value)
Cytosolic ribosome	171	43 (3.6 × 10^-26^)	54 (3.2 × 10^-29^)	17 (9.4 × 10^-16^)
Ribosome	284	51 (1.1 × 10^-23^)	67 (8.6 × 10^-28^)	21 (6.8 × 10^-16^)

Genes bound by Gcn5p (225 genes), Sas3p (335 genes) or both (47 genes) with a binding ratio ≥1.5 were classified on the basis of over-represented attributes using the FuncAssociate web tool [52]. The number of genes bound for every category, the number of overall genes with this attribute and *P *values are shown. Only the two categories with the most significant *P *values are shown. The search for GO categories was done using FuncAssociate [52].

### There is no correlation between Sas2p, Hpa2p and Nut1p binding and transcription rate

For the other HATs analyzed, the lack of correlation obtained in the case of Hpa2p or Sas2p can be explained assuming that these proteins are not general activators. In fact, if we consider a threshold binding ratio of 1.5, there are 378 and 916 genes that can be considered as bound by Hpa2p and Sas2p, respectively, and these genes correspond to genes that are not intensely transcribed (results not shown). Interestingly, when the threshold of the binding ratio is increased to 2, there are 252 genes bound by Sas2p, 13 of them belonging to categories including double-strand break repair or ubiquitin-specific protease activity (Table 3). This is the first relationship described between Sas2p and these processes. Sas2p has been implicated in telomere silencing through acetylation of Lys16 of H4 [40]. However, we did not detect any identifiable pattern of chromosomal distribution for the binding targets of this protein. In the case of Elp3p, although a correlation between transcription rate and its binding ratio can be observed (Figure 2), it was very low. Moreover, the genes bound by Elp3p with a binding ratio ≥1.5 do not cluster in any GO category. Therefore, we cannot extract any conclusion about the genome-wide location of Elp3p. On the other hand, it is intriguing that the genome occupancy of Nut1p, a component of Mediator, does not correlate with transcription rate. One possible explanation is that cross-linking of Nut1p with DNA was prevented by the complexity of Mediator, a multiprotein complex formed by 21 subunits in yeast [41]. Furthermore, the genes bound by Nut1p with a binding ratio ≥1.5 do not cluster in any GO category (results not shown).

**Table 3:** Genes bound by Sas2p

GO attribute	No. of genes in category	No. of genes bound by Sas2p (*P *value)	Genes
Non-recombinational repair	24	7 (2.5 x 10^-5^)	*RAD50*, *YKU70*, *SIR3*, *SIN3*, *LIF1*, *POL4*, *RAD1*
Ubiquitin-specific protease activity	17	6 (2.8 x 10^-5^)	*UBP2*, *UBP3*, *UBP6*, *UBP7*, *UBP10*, *UBP12*

Genes bound by Sas2p with a binding ratio ≥2.0 (252 genes) were classified on the basis of over-represented attributes using the FuncAssociate web tool [52]. The number of genes bound for every category, the number of overall genes with this attribute, *P *values and the names of the identified genes are shown. Only the two categories with the most significant *P *values are shown. The search for GO categories was done using the FuncAssociate server [52].

### *In vivo *H3K14 acetylation by Gcn5p and Sas3p HATs

**Figure 4:**
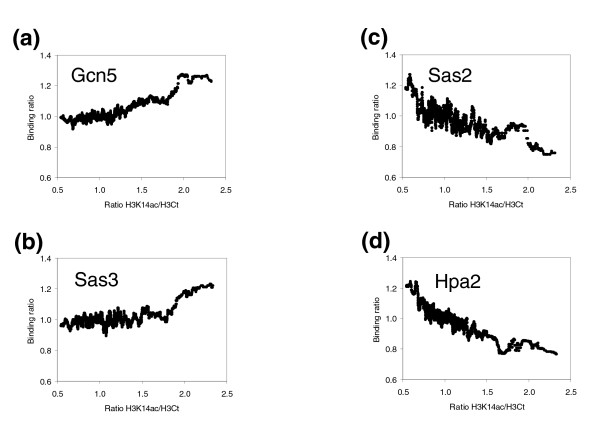
Correlation between Gcn5p, Sas3p, Sas2 and Hpa2 occupancies and acetylated H3K14 enriched regions. Occupancies from **(a) **Gcn5p, **(b) **Sas3p, **(c) **Sas2p and **(d) **Hpa2p were calculated as in Figure 2. Acetylation at Lys14 of histone H3 was obtained by two ChIP-chip experiments. The first was performed by immunoprecipitating WCE with an antibody that recognizes the carboxyl termini of histone H3 (α-H3Ct) and the other one by immunoprecipitating with α-K14acH3. Ratios from the α-K14acH3 ChIP-chip experiment and the control from the α-H3Ct ChIP-chip experiment were calculated, and their moving median was obtained over a sliding window of 100 ORFs ordered by Gcn5p, Sas3p, Sas2p or Hpa2 occupancy.

Considering that Gcn5p and Sas3p are HATs whose main target *in vitro *is H3K14, we focused our study on this modification. To determine the involvement of these proteins in the *in vivo *acetylation state of H3K14, it was necessary to test if our macroarray method could be utilized to assess this specific modification. To achieve this goal, we examined a previously reported positive correlation between H3K14ac and transcriptional activity [24]. Our results also showed a positive correlation between transcriptional activity of genes and their H3K14ac state. Furthermore, this positive correlation is observed only when the histone H3 carboxy-terminal antibody is used as a control (Additional data file 8). Next, we tested whether Sas3p or Gcn5p binding to chromatin is related to H3K14ac levels. In agreement with previously reported results [24], Figure 4a shows a positive correlation between Gcn5p occupancy and the modification known to be catalyzed by this enzyme *in vitro*. Figure 4b shows that this positive correlation is also evident for Sas3p, albeit less pronounced than for Gcn5p. These results strongly suggest that both HATs acetylate H3K14 *in vivo*. We have performed the same study on H3 acetylating HATs Sas2p and Hpa2p and we obtained a negative correlation in both cases (Figure 4c,d, respectively). A possible explanation for these unexpected results is that these enzymes are not able to acetylate H3K14 *in vivo*. In any case, it seems clear that the acetylation level of H3K14 at genome regions bound by Sas2p and Hpa2p is lower than that of the rest of the chromatin. It is still unknown which HATs are responsible for the high level of acetylation of H3K14, but our data suggest that these enzymes may be Gcn5p and, to a lesser extent, Sas3p.

Sas3p is the catalytic subunit of NuA3, a complex identified on the basis of its ability to preferentially acetylate Lys14 and, to a lesser extent, Lys23 of nucleosome-bound histone H3 *in vitro *[39,42]. Moreover, it has recently been reported that Yng1, a component of NuA3, interacts directly with K4 trimethylated H3 amino-terminal tail (H3K14me3) and that this interaction enhances *in vitro *H3K14-specific NuA3 HAT activity [43]. Furthermore, mutation of H3K14 to arginine phenocopies the *sas3*Δ phenotype [44]. All these findings strongly suggest that Sas3p acetylates H3K14 *in vivo*. On the other hand, whereas the preference of Gcn5p for H3K14 *in vitro *is well-documented [12-14], the role of this HAT in the acetylation of this position *in vivo *is not well known. It is surprising that ten years after the identification of Gcn5p as a HAT, its role on the *in vivo *acetylation of H3K14 remains unclear. Thus, it has been reported that Gcn5p acetylates H3 lysines 9, 18, 23 and 27 but not Lys14 *in vivo *[22,23]. In contrast, a genome-wide positive association between Gcn5p occupancy, acetylation of H3K14 and transcriptional activity has also been demonstrated [24].

**Figure 5:**
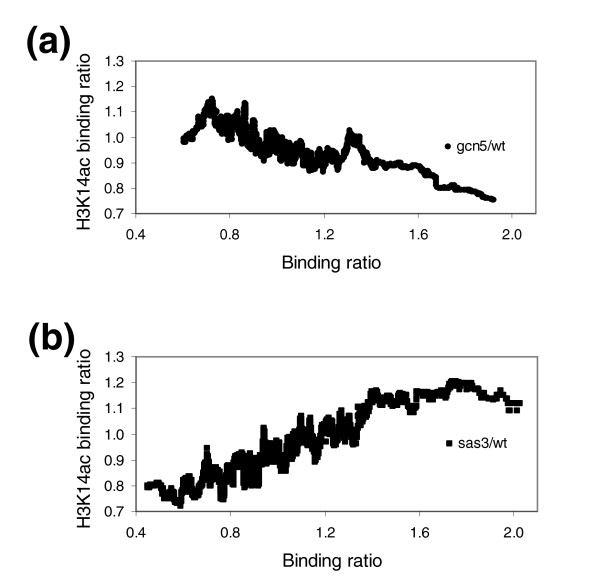
Correlation between Gcn5p and Sas3p occupancies and acetylation of K14H3 in *gcn5*Δ or *sas3*Δ strains. Correlation between **(a) **Gcn5p and **(b) **Sas3p occupancies and acetylated K14H3 enriched regions in their respective deletion mutants. Occupancies from Gcn5p and Sas3p were calculated as in Figure 4, and the same was done for acetylated regions at *gcn5*Δ or *sas3*Δ.

To expand upon these observations, we analyzed the genome-wide effect of the loss of *GCN5 *or *SAS3 *on the H3K14ac state. We plotted the change of H3K14 acetylation in genomic regions of mutant strains as a function of their protein occupancy using epitope-tagged strains. We found a correlation between the decrease in H3K14 acetylation and the Gcn5p genome occupancy (Figure 5a; Additional data file 9). This finding represents the first evidence that Gcn5p mediates the *in vivo *acetylation of the same H3 lysine position, which is its major target *in vitro*. Obviously, we cannot exclude the possibility that Gcn5p also catalyzes acetylation at other positions not analyzed here *in vivo*. Surprisingly, we observed a contrary effect in *sas3*Δ strains (Figure 5b; Additional data file 10); the higher the binding ratio of Sas3p, the higher the H3K14ac state in the *sas3*Δ mutant with respect to the wild-type strain. This increase persisted until binding ratios of 1.5. From Figure 5b it can be deduced that Sas3p should actually be an inhibitor of H3K14ac. This result is difficult to interpret but a possible explanation is that, in the absence of Sas3p, another HAT with H3K14 specificity, perhaps Gcn5p, acetylates this position. If this assumption is correct, in wild-type strains, Sas3p prevents Gcn5p from accessing potential H3K14 targets.

The representation of Figure 5 depicts the complete binding ratio data set for Sas3p and Gcn5p. This strategy of data representation has been successfully used by others [8,24,44-46] because it easily identifies general trends. However, the measure of significance level for each ORF using a z-test for independent data is another way of analyzing the results. This type of analysis yielded a limited number of significant genes (see Materials and methods). Application of this statistical test to the results obtained in the analysis of the relative levels of H3K14ac in the *sas3*Δ mutant identified a set of 434 genes presenting significantly lower acetylation states. Interestingly, when we examined the GO categories of these genes, several categories were over-represented, almost all of them involved in cell division processes (Table 4). The significance of the clustering observed suggests that Sas3p may be implicated in this process *in vivo*, possibly through the acetylation of H3K14. In this sense, previous reports show that *gcn5*Δ strains, carrying a temperature-sensitive allele of *SAS3 *mutants, arrest in the G_2_/M phase of the cell cycle when grown at restrictive temperature [15]. This phenotype is, however, *gcn5*Δ dependent. While Sas3p has been directly implicated in several important processes such as chromatin silencing at telomeres and silencing of the mating-type cassette, the findings shown here are, to our knowledge, the first direct relationship described between Sas3p and cellular division.

**Table 4:** Genes that showed a significantly decreased H3K14 acetylation level in the *sas3*Δ mutant

GO attribute	No. of genes in category	No. of genes	*P *value
Cell cycle/cell-division cycle	397	54	2.8 × 10^-7^
Mitotic cell cycle	233	37	5.8 × 10^-7^
Cell budding/budding	77	18	2.2 × 10^-6^
Development	365	48	3.7 × 10^-6^
Reproduction	251	36	9.5 × 10^-6^
Plasma membrane/bacterial inner membrane/cell membrane/cytoplasmic membrane/juxtamembrane/Plasmalemma	232	34	1.1 × 10^-6^
Cell organization and biogenesis	1,149	111	1.6 × 10^-5^
Cytoskeleton organization and biogenesis	288	39	1.6 × 10^-5^

Groups of genes that showed a significantly decreased H3K14ac level in the *sas3*Δ mutant relative to the wild-type strain (434 genes) were classified based on over-represented attributes using the FuncAssociate web tool [52]. The number of genes bound for every category, the number of overall genes with this attribute and *P *values are shown. Only the eight categories with the most significant *P *values are shown. The search for GO categories was done using the FuncAssociate server [52].

## Conclusion

We have developed a new ChIP-chip method based on the use of ORF macroarrays that can be reutilized several times and, therefore, applied to several samples. The methodology of ChIP-chip described allows for the rapid scanning of HAT localization of chromatin binding proteins in yeast and could represent a low-cost alternative to microarrays. Nevertheless, the methodology described here has several limitations. For instance, with this macroarray, which contains entire ORF sequences as probes, it is clearly impossible to distinguish between IG binding sites and those within an ORF. Binding sites within large IGRs will also be undetectable. On the other hand, promoter binding detected by this method will be biased towards sites that are closest to an ORF and we cannot discard the possibility that this represents a functionally distinct subset of promoters. Finally, probes prepared from very long ORFs (longer than 3 kb) will work poorly for detection of promoter regions and detection of protein binding will weaken as ORF length increases [26]. However, these probes represent only 7% of the total. Despite these limitations, in this report we have demonstrated that macroarrays can reproduce the same results previously obtained by other researchers using microarrays.

Most of the HATs present in yeast exhibit HAT activity *in vitro *towards histone H3 and, especially, to H3K14. In this study, we have used our methodology to investigate the genome-wide association of known *S. cerevisiae *HATs that have shown specificity for this residue. Considering that there are at least 12 lysines that could act as possible acetylation sites in the yeast inner histones, H3, H4, H2A and H2B, we think that this preferential specificity for H3K14 may be of particular importance. For instance, this position may have a central role in the stability of the nucleosome. Thus, this specific modification of this H3 residue may directly facilitate the action of the transcriptional apparatus and other chromatin functions. Among the HATs analyzed, genome-wide occupancy of Gcn5p and Sas3p correlates with the transcriptional rate of the genes, whereas in the case of Elp3p, Sas2p, Hpa2p and Nut1p, no significant correlation was observed. These results demonstrate that Sas3p, a protein initially described as implicated in transcriptional silencing, is recruited to intensely transcribed genes, similar to what has been observed for Gcn5p. In this sense, it has recently been proposed that NuA3 is targeted to and/or retained at sites of H3K4me3 through interaction with Yng1, promoting H3K14ac, positively regulating downstream transcription events [43]. The observation that Sas3p and Gcn5p HATs are recruited to similar active genes also supports the hypothesis [15] that Gcn5p and Sas3p have redundant functions. Moreover, we obtained a positive correlation between Sas3p (and Gcn5p) binding and the acetylation state of H3K14. The observation that Sas3p and Gcn5p are related to processes such as protein biosynthesis or cell division, in which an intense transcriptional activity is required, supports the hypothesis that H3K14ac plays a role in facilitating transcription. These findings strongly suggest that Gcn5p and Sas3p acetylate H3K14 *in vivo*. However, analysis of the genome-wide effect of the loss of *GCN5 *or *SAS3 *on the H3K14ac state may be subject to different interpretations. Thus, the negative correlation found between H3K14ac in the *gcn5*Δ strain and the Gcn5p occupancy using an epitope-tagged strain provides good evidence that Gcn5p acetylates this residue *in vivo*. On the contrary, the positive correlation found between H3K14ac in the *sas3*Δ strain and the Sas3p occupancy in the epitope-tagged strain suggests that Sas3p occupancy has a negative influence on the H3K14ac state. This striking result can be interpreted by considering that there is another HAT *in vivo *with H3K14 specificity that acetylates intensely this position *in vivo *in the absence of Sas3p. Based on the results shown here, we suggest that this HAT is Gcn5p. In any case, these results show implicitly that, *in vivo*, different HATs with overlapping specificities compete for chromatin binding sites and that this competition can play an important role in the regulation of the establishment of the histone code signals. Thus, one possible role of Sas3p could be to displace Gcn5p from certain key sites.

## Materials and methods

### Yeast strains and genetic methods

*S. cerevisiae *strains for genome-wide location analysis used in this study are listed in Table 5. Genomic ORFs were tagged or deleted using a PCR-based strategy [47,48]. Epitope tags were inserted at carboxyl termini by targeted integration. DNA fragments, with flanking sequences homologous to the desired gene, were amplified from the appropriate template plasmid [48-50] (a gift from PM Alepuz). Correct homologous integration was verified by PCR. Transformants were also assayed for expression of the desired tagged protein by western blotting.

**Table 5:** Strains used in this study

Strain	Genotype	Source
YPH250	MATa, *ura3-52, lys2-801*, *ade2-101*,*trp1-Δ1*,*his3-Δ200, leu2Δ1*	[53]
MW671	MATa, *ura3-52, lys2-801, ade2-101, trp1-Δ63*,*his3-Δ200*, *leu2Δ1*, *rpc160Δ*1::*HIS3*, pC160-240 (*TRP1 *CEN4 HA3-*RPC160*)	[54]
BY4742	MATα, *ura3Δ0, lys2Δ0, his3Δ1, leu2Δ0*	[55]
BQS1350	BY4742 *GCN5*-MYC13-KanMX6	This work
BQS1217	BY4742 *SAS3*-HA6-*HIS3*	This work
BQS1218	BY4742 *HPA2*-HA6-*HIS3*	This work
BQS1197	BY4742 *NUT1*-HA6-*HIS3*	This work
BQS1343	BY4742 *ELP3*-HA6-*HIS3*	This work
BQS1451	BY4742 *SAS2*-HA6-*HIS3*	This work
BQS1181	BY4742 *gcn5Δ::KanMX4*	This work
BQS1389	BY4742 *sas3Δ::KanMX4*	This work

### Chromatin immunoprecipitation

The targets of catalytic HAT subunits and the Rpc160 subunit of RNA Pol III from yeast were identified by combining chromatin immunoprecipitation and ORF macroarray hybridization. We designed a specific protocol for ORF macroarrays based on a previously described method originally devised for intergenic microarrays [51].

Three independent cultures from tagged and control strains were grown. A volume of 40 ml YPD (1% yeast extract, 2% peptone, 2% glucose) culture from each one (OD 600 ≈ 0.5-0.8) was set aside, and proteins were cross-linked to their target sites *in vivo *by adding formaldehyde to a final concentration of 1%. Cells were incubated for 30 minutes at room temperature with occasional mixing, and cross-linking was quenched by adding glycine to a final concentration of 125 mM. Cells were washed three times with 30 ml of ice-cold phosphate-buffered saline buffer (140 mM NaCl, 2.7 mM KCl, 10 mM Na_2_HPO_4_, 1.8 mM KH_2_PO_4_, pH 7.4). Pelleted cells were frozen after this step. Cells were thawed on ice and resuspended in 300 μl lysis buffer (50 mM HEPES-KOH pH 7.5, 140 mM NaCl, 1 mM EDTA, 1% Triton X-100, 0.1% sodium deoxycholate, 1 mM PMSF (Phenylmethylsulfonyl fluoride), 1 mM benzamidine and 1 pill of protease inhibitor cocktail (Roche Diagnostics, Mannheim, Germany) dissolved in every 25 ml of buffer). The equivalent of 0.2 ml of frozen glass beads (425-600 mm; Sigma-Aldrich, St Louis, MO, USA) was added to the cellular suspension and cells were lysed at 4°C by 25 minutes of vortexing in a Genie 2 vortex with Turbo mix at maximum power. Lysis buffer (400 μl) was added and the extract was transferred to a new tube. Sonication of DNA was performed in order to obtain DNA fragments between 400 and 3,000 bp, with an average size of 800 bp. Chromatin was sonicated on ice, 3 pulses of 10 s at 38% amplitude in a Vibracell VCX500 (Sonics&Materials, INC., Newtown, CT, USA). Cell debris was removed by centrifugation at 13,000 rpm at 4°C for 10 minutes. An aliquot of 50 μl of this whole cell extract (WCE) was kept to check the quality and size of the isolated chromatin.

The antibodies used were 2 μg of rat anti-HA 3F10 (Roche), 20 μg of mouse anti-MYC 9E11 (Roche), 15 μl of rabbit α-K14acH3 chip grade (Upstate, Lake Placid, NY, USA) and 20 μg of rabbit α-H3Ct (Abcam Inc., Cambridge, UK). Rat and mouse antibody complexes were collected using 100 μl of a 50% (v/v) suspension of Protein G Sepharose 4FF (Amersham Biosciences, Piscataway, NJ, USA) equilibrated in 5 mg/ml bovine serum albumin (BSA) lysis buffer. Rabbit antibody complexes were collected using 50 μl of a suspension of sheep α-rabbit IgG M-280 dynabeads (Dynal, Invitrogen Corp., Carlsbad, CA, USA) in a final volume of 120 μl. Suspensions were incubated overnight at 4°C. DNA fragments specifically cross-linked to the epitope-tagged protein were purified by immunoprecipitation with the indicated monoclonal antibodies coupled to Protein G Sepharose for 4 h at 4°C. We included control immunoprecipitations of lysates from wild-type strains with α-HA, α-MYC or α-H3Ct antibodies. Beads were washed twice with lysis buffer, twice with 500 mM NaCl lysis buffer, twice with wash buffer (10 mM Tris-HCl pH 8.0, 250 mM LiCl, 0.5% Nonidet P-40, 0.5% sodium deoxycholate, 1 mM EDTA pH 8.0, 1 mM PMSF, 1 mM benzamidine and 1 pill of protease inhibitor cocktail/25 mL (Roche)), once with TE (10 mM Tris-HCl, pH 8.0, 1 mM EDTA) containing 1 mM PMSF, and finally collected. Two successive elutions were performed with 100 and 150 μl of elution buffer (50 mM Tris-HCl pH 8.0, 10 mM EDTA, 1% SDS) by incubating for 10 minutes at 65°C each time. The eluted fraction of protein cross-linked to DNA was treated overnight at 65°C to reverse the cross-linking. For WCE, 200 μl of elution buffer were added before reversal of cross-linking (from 1 h to overnight).

### DNA purification and annealed linker ligation

Proteins were degraded by adding 50 μg of proteinase K and SDS to 2.5% final concentration, and incubation at 37°C for 1 h. DNA was purified by phenol/chloroform/isoamylic alcohol extraction. For WCE, the aqueous phase was directly ethanol precipitated, resuspended in 15 μl of milliQ water, and treated for 30 minutes with 10 μg of RNAse A. The WCE chromatin was visualized by agarose electrophoresis to check the quantity and size of the fragments. For immunoprecipitated DNA samples, the aqueous phase was subsequently purified with Montage PCR columns (Millipore Corp., Billerica, MA, USA). The total eluted volume was treated with 10 μg of RNAse A for 30 minutes at 37°C. Ethanol precipitation in the presence of 20 μg of glycogen as a carrier was performed. The immunoprecipitated DNA was blunted by T4 phage DNA polymerase in a reaction volume of 124 μL (T4 DNA Pol buffer, 40 μg/μl BSA, 80 μM dNTPs, 0.6 U T4 DNA Polymerase from Roche). The reaction was allowed to proceed for 20 minutes at 12°C. After phenol/chloroform/isoamylic alcohol extraction, DNA was ethanol-precipitated in the presence of 11 μg of glycogen and was ligated in a final volume of 50 μl with annealed linkers oJW102 and oJW103 (1.5 μM of each primer) [24]. The reaction was carried out overnight at 16°C and ligated DNA was precipitated and resuspended in 25 μl milliQ water.

### DNA amplification and macroarray hybridization

Ligation Mediated PCR [51] was used for DNA amplification. Briefly, ligated DNA was dissolved in final volume of 40 μl (1× Biotaq buffer (from Bioline, London, UK), 2 mM MgCl_2_, 0.25 mM dNTPs, 1.25 μM oligonucleotide oJW102). The reaction was started by incubating for 2 minutes at 55°C and paused to add 10 μl of reaction mix (1× Biotaq buffer, 2 mM MgCl_2 _and 5 U BioTaq from Bioline). The program was resumed with 5 minutes at 72°C, 2 minutes at 95°C and 33 cycles of 30 s at 95°C, 30 s at 55°C and 2 minutes at 72°C. A 5 μl DNA aliquot of the LM-PCR was analyzed on a 1.2% agarose gel (to check for a smear from 200-2,500 bp and an average size of 600 bp), and the rest was purified with Montage PCR columns. DNA was precipitated overnight and resuspended in 25 μl of milliQ water. The concentration of nucleic acids was determined using a spectrophotometer.

The PCR product was used as a template for the radioactive labeling reaction. This reaction consisted of a single amplification step where Taq polymerase was incubated with a mix of ^33^P-dCTP, cold dCTP and the rest of dNTPs. The choice of the ratio between hot and cold dCTP is essential to produce long-sized DNA samples. Both Klenow and Taq polymerase were assayed in the presence of different radioactive/cold ratios. The labeling yield was much higher for Taq polymerase at any of the cold dCTP concentrations assayed (results not shown). Increases in the concentration of cold dCTP produced longer sizes of labeled DNA, although the incorporation of ^33^P-dCTP was reduced. We chose a ratio that produced DNA products with an average size of 600 bp and an acceptable threshold of radioactive labeling. The reaction mixture contained 1.5 μg of amplified DNA in 50 μl (1× Biotaq buffer, 2 mM MgCl_2_, 0.2 mM dATP, dTTP and dGTP, 25 μM dCTP, 1 μM oJW102, 0.8 μCi α-^33^P-dCTP and 5 U Biotaq). The mix was denatured for 5 minutes at 95°C, annealed 5 minutes at 50°C and was amplified during 35 minutes at 72°C. The reaction product was purified through a ProbeQuant G-50 column (Amersham Biosciences) to remove unincorporated ^33^P-dCTP and oligonucleotides.

The macroarrays used contained PCR products representing full-length ORFs for 6,049 genes of *S. cerevisiae *[26] (Servicio de Chips de DNA, Universitat de València). After pre-hybridizing macroarrays for 1 h in hybridization solution (5× SSC, 5× Denhart's, 0.5% SDS) at 65°C in a rotator oven, hybridizations were performed using the same solution containing labeled denatured DNA (3 × 10^6 ^dpm/ml) for 16-18 h. After hybridization, the membranes were washed once in 2× SSC, 0.1% SDS for 20 minutes, and twice in 0.2× SSC, 0.1% SDS for 30 minutes. They were exposed to an imaging plate (BAS-MP, Fujifilm) for 48-72 h and read in a PhosphorImager (FLA-3000, Fujifilm) at 50 μm resolution. After analysis of macroarray filters, they were stripped as described previously [26] and hybridized with an appropriate sample under the same conditions.

### Image analysis and data processing

Images were quantified by using ArrayVision 7.0 software (Imaging Research, Inc., GE Healthcare Europe, Barcelona, Spain). The signal intensity for each spot was the background subtracted ARM Density (artifact removed median). Only values 1.5 times over the corresponding background were taken as valid measurements. Reproducibility of the replicates was checked using ArrayStat software (Imaging Research, Inc.). We considered data as independent and used a minimum number of two valid replicates in order to calculate the mean and standard deviation values for every gene. Normalization between conditions was done using the global median method. In order to detect the statistically significant, immunoprecipitated genes for condition, a z-test for independent data was applied and, consequently, a z-score for every gene was obtained. A *P *value of 0.05 and the false discovery rate method were used for controlling the false positive overall error rate. The ratio between the two conditions considered in each experiment, after normalization, was taken as the binding ratio.

## Additional data files

The following additional data are available with the online version of this paper. Additional data file 1 is a table listing the binding ratio of Rpc-160-HA, a subunit of RNA polymerase III from *S. cerevisiae*, in a ChIP-chip experiment using anti-HA antibodies (see Materials and methods). The binding ratio is calculated as the ratio of chromatin-immunoprecipitated signals between the epitope-tagged strain and the un-tagged parent, using the same antibodies. Additional data files 2 to 7 are tables listing the binding ratio of Sas3-HA, Gcn5-MYC, Elp3-HA, Nut1-HA, Sas2-HA and Hpa2-HA, respectively, in ChIP-chip experiments using anti-HA or anti-MYC antibodies. The binding ratio was calculated as described in Additional data file 1. Additional data file 8 is a table listing the acetylated H3K14 state of the yeast genes. Acetylation at H3K14 was obtained by two ChIP-chip experiments. The first was performed by immunoprecipitating WCE with an antibody that recognizes the carboxyl termini of histone H3 (α-H3Ct) and the other one by immunoprecipitating with α-K14acH3. Ratios from the α-K14acH3 ChIP-chip experiment and the control from the α-H3Ct ChIP-chip experiment were calculated and are listed. Additional data files 9 and 10 are tables listing the acetylated H3K14 state of the yeast genes in *gcn5*Δ and *sas3*Δ strains, respectively. Acetylation at H3K14 was obtained as described in Additional data file 8.

## Supplementary Material

Additional data file 1 Binding ratio of Rpc-160-HA, a subunit of RNA polymerase III from *S. cerevisiae*, in a ChIP-chip experiment using anti-HA antibodies (see Materials and methods). The binding ratio was calculated as the ratio of chromatin-immunoprecipitated signals between the epitope-tagged strain and the un-tagged parent, using the same antibodies. Click here for file

Additional data file 2 The binding ratio was calculated as the ratio of chromatin-immunoprecipitated signals between the epitope-tagged strain and the un-tagged parent, using the same antibodies. Click here for file

Additional data file 3 The binding ratio was calculated as the ratio of chromatin-immunoprecipitated signals between the epitope-tagged strain and the un-tagged parent, using the same antibodies. Click here for file

Additional data file 4 The binding ratio was calculated as the ratio of chromatin-immunoprecipitated signals between the epitope-tagged strain and the un-tagged parent, using the same antibodies. Click here for file

Additional data file 5 The binding ratio was calculated as the ratio of chromatin-immunoprecipitated signals between the epitope-tagged strain and the un-tagged parent, using the same antibodies. Click here for file

Additional data file 6 The binding ratio was calculated as the ratio of chromatin-immunoprecipitated signals between the epitope-tagged strain and the un-tagged parent, using the same antibodies. Click here for file

Additional data file 7 The binding ratio was calculated as the ratio of chromatin-immunoprecipitated signals between the epitope-tagged strain and the un-tagged parent, using the same antibodies. Click here for file

Additional data file 8 Acetylation at H3K14 was obtained by two ChIP-chip experiments. The first was performed by immunoprecipitating WCE with an antibody that recognizes the carboxyl termini of histone H3 (α-H3Ct) and the other one by immunoprecipitating with α-K14acH3. Ratios from the α-K14acH3 ChIP-chip experiment and the control from the α-H3Ct ChIP-chip experiment were calculated and are listed. Click here for file

Additional data file 9 Acetylation at H3K14 was obtained by two ChIP-chip experiments. The first was performed by immunoprecipitating WCE with an antibody that recognizes the carboxyl termini of histone H3 (α-H3Ct) and the other one by immunoprecipitating with α-K14acH3. Ratios from the α-K14acH3 ChIP-chip experiment and the control from the α-H3Ct ChIP-chip experiment were calculated and are listed. Click here for file

Additional data file 10 Acetylation at H3K14 was obtained by two ChIP-chip experiments. The first was performed by immunoprecipitating WCE with an antibody that recognizes the carboxyl termini of histone H3 (α-H3Ct) and the other one by immunoprecipitating with α-K14acH3. Ratios from the α-K14acH3 ChIP-chip experiment and the control from the α-H3Ct ChIP-chip experiment were calculated and are listed. Click here for file
